# Towards decent work in the digital age: introducing the fairwork project in Germany

**DOI:** 10.1007/s41449-021-00247-w

**Published:** 2021-06-07

**Authors:** Alessio Bertolini, Maren Borkert, Fabian Ferrari, Mark Graham

**Affiliations:** 1 Oxford Internet Institute, University of Oxford, 1 St Giles’, Oxford OX1 3JS, UK; 2 XU Exponential University, August-Bebel-Str. 26–53, 14482 Potsdam, Germany

**Keywords:** Decent work, Platform economy, Digital business models, Fairness ranking, Covid-19, Menschenwürdige Arbeit, Plattformökonomie, Digitale Geschäftsmodelle, Fairness-Ranking, Covid-19

## Abstract

The Fairwork Project is an international action-research project that currently operates in over 20 countries. The project focuses on working conditions in the platform economy, in order to develop ‘fairness ratings’ for digital labour platforms. With respect to Germany, the project evaluated the working conditions offered by ten digital labour platforms, by scoring them against the Fairwork principles and producing a national league table. We found that even in a highly regulated labour market context like the German one, platform workers experience precarity and insecurity and have limited access to employment rights. A number of platform workers are classified as employees rather than self-employed, and this guarantees a number of employment rights, including entitlement to minimum wage, health and safety protection and social protection. However, the existence of an employment relationship does not necessarily ensure platform work to be fair as other factors, including the existence of complex networks of subcontracting, erode labour standards and deprive workers of basic employment rights.

*Practical Relevance: *While there are tens of millions of digital platform workers around the world performing functions essential to society—as demonstrated drastically by the Covid-19 pandemic—by supplying food, care and passenger transportation services, many platform workers face low pay, precarity as well as poor and dangerous working conditions. Exposing fracture lines of inequalities affecting particularly women, migrants and minority-ethnic groups who form the core part of the gig workforce, the international Fairwork research project aims not just to understand the gig economy, but to change it.

## Abstract (de)

Das Fairwork-Projekt ist ein internationales Aktionsforschungsprojekt, das derzeit in über 20 Ländern stattfindet. Das Projekt fokussiert auf die Arbeitsbedingungen in der aktuellen Plattformökonomie und entwickelt jährliche „Fairness-Ratings“ für digitale Arbeitsplattformen. Für Deutschland hat das Projekt die Arbeitsbedingungen von zehn digitalen Arbeitsplattformen evaluiert, indem es sie anhand der Fairwork-Prinzipien bewertete und eine nationale Rangliste erstellte. Wir fanden heraus, dass selbst in einem stark regulierten Arbeitsmarktkontext wie dem deutschen, Plattformarbeiter Prekarität und Unsicherheit erleben und nur begrenzten Zugang zu Arbeitsrechten haben. Eine Reihe von Plattformarbeiter*innen wird als Arbeitnehmer*innen und nicht als Selbstständige eingestuft, was eine Reihe von Arbeitsrechten garantiert, darunter den Anspruch auf Mindestlohn, Gesundheits- und Sicherheitsschutz sowie soziale Absicherung. Das Vorhandensein eines Arbeitsverhältnisses garantiert jedoch nicht unbedingt, dass Plattformarbeit fair ist, da andere Faktoren, einschließlich der Existenz komplexer Netzwerke von Unterauftragnehmer*innen, die Arbeitsstandards aushöhlen und den Arbeitnehmer*innen grundlegende Beschäftigungsrechte vorenthalten.

*Praktische Relevanz: *Während es weltweit Millionen digitaler Plattformarbeiter*innen gibt, die für die Gesellschaft unverzichtbare Funktionen erfüllen – wie die Covid-19-Pandemie drastisch gezeigt hat –, indem sie Lebensmittel, Pflege- und Personenbeförderungsdienste bereitstellen, sind viele von ihnen mit niedriger Bezahlung, Prekarität sowie schlechten und gefährlichen Arbeitsbedingungen konfrontiert. Das internationale Fairwork-Forschungsprojekt, das Bruchlinien der Ungleichheit aufzeigt, von denen insbesondere Frauen und Migrant*innen betroffen sind, die den Kern der Gigworkforce ausmachen, zielt darauf ab, die Plattformökonomie nicht nur zu verstehen, sondern sie zu verändern.

## Introduction

Germany strongly benefits from its embeddedness in the global economy (Jungbluth 2019). Yet, megatrends such as globalisation, digitalisation and demographic change may as well pose serious challenges to people’s employment and income equalities in the country (Petersen and Steiner 2019). Within this context, two recent legislative actions taken by the German government in 2021 are of particular interest for assuring fair work standards in today’s economy, ie. the ‘Law on Modernization of the Passenger Transportation Act (PBefG)’ and the ‘Law on Due Diligence in Supply Chains’ (BMVI 2021; BMZ 2021). Both legislative initiatives can be understood as attempts by German ministries to create fair conditions for business activities spurred by globalisation and digitalisation, which have determined our everyday life for years.

With the amendment to the Passenger Transportation Act (PBefG) and the ‘Law on Due Diligence in Supply Chains’, the German government seeks to set a legal basis for fair working conditions in new digital mobility offers/services and globalised supply and value chains (BMVI 2021). According to the German Federal Ministry for Economic Cooperation and Development (BMZ), too few German companies voluntarily fulfill their due diligence obligations along the entire supply and value chain—also due to the fact that the processes and technologies used are outdated (BMZ 2021). Digitalisation, properly implemented, may act as a lever here for fairer global trade as it facilitates transparency across the entire supply and value chain.

Against the backdrop of these political developments, we introduce the Fairwork project: an international action-research project that currently operates in the UK, Germany, Austria, South Africa, India, Chile, Ecuador, Brazil, Serbia, Ukraine, the United States, Ghana, Kenya, Colombia, Belgium, Argentina, Indonesia, Hong Kong, Egypt, and Bangladesh. The project focuses on working conditions in the platform economy, in order to develop ‘fairness ratings’ for digital labour platforms. It makes use of these ratings as a lever to improve pay and conditions for platform workers, through media and consumer pressure. Fairwork’s research agenda intends not just to understand the gig economy, but to change it. By bringing workers, platforms, scholars, labour researchers and policymakers to the table, Fairwork researchers work to collaboratively develop and embed principles of fair work into the script of the gig economy.

We first set the stage for Fairwork’s research in Germany by discussing key characteristics the country’s platform economy. We then outline the Fairwork methodology and present our results. We conclude by shedding a light on the policy implications of the Fairwork approach.

## Germany’s platform economy

Although there is no all-encompassing empirical evidence on the precise number of workers on digital labour platforms in Germany, estimates range from 500,000 to 1.6 million workers depending on the method of counting (BMAS 2018). The majority of them work in household-related services (e.g. cleaning services, pet sitting, care work), logistics (e.g. delivery and courier services) and transport (e.g. driving services) (Pesole et al. 2018). Numerous platform companies have been founded in Berlin, including Helpling, Delivery Hero and Betreut.de. In recent years, the regulation of the platform economy is clearly becoming a key priority for politicians and regulators. Key institutions include the Federal Ministry of Transport and Digital Infrastructure (BMVI), the Federal Ministry for Work and Social Affairs (BMAS) and various parliamentary committees on the national and regional levels.

At the domestic level, for years digital platforms have been offering services for e.g. passenger transportation in German cities, by providing rides to licensed car rental companies (like the internationally operating firm Uber) or through collective journeys (pooling) like Clevershuttle or Moia (both domestic startups)—although operating in a legal grey area: while the ride pooling services Clevershuttle and Moia operated largely on the legal basis of a so-called experimentation clause, Uber had to adjust its business model several times due to violations of existing German law (DPA 2015). Uber’s Taxi services, for example, which brokers locally licensed cabs to customers, is competing with other brokerage service cab apps like myTaxi and provoking fierce resistance and criticism by local taxi associations calling for an Uber-ban on spontaneous trips in major cities (t3N 2020).

The German labour market is characterized by a long tradition of tripartite social partnership between three institutional pillars: the government, labour unions and employers’ associations. As Thelen (2018) argues, Germany’s historic legacy of tripartite social partnership between employers ‘organizations, workers’ organizations and the government, as well as its strict labor regulations, provide a framework of strong institutional scrutiny and balance against the uncertainty and precarity created by digital labour platforms. In contrast to a range of other countries where the Fairwork project conducts empirical research, many platform workers in Germany are classified as employees rather than as independent contractors, notably those working for the food-delivery quasi-monopolist Lieferando (Handke 2020).

Yet even in a highly regulated labour market context like Germany, digital labour platforms find ways to exacerbate the precarity of workers, even in cases where they are provided with employment status. The findings of our pilot study published in 2020 indicate that Germany’s relatively stringent labour regulations provide a number of basic protections for workers, but do not necessarily bring working conditions in the platform economy into being fair (Fairwork 2020a).

This discrepancy echoes the wider tendency that the German labour market is increasingly reliant on low-wage, precarious migrant labour. In 2018, more than half of the working population were engaged in employment relationships that were subject to social insurance contributions, with an unemployment rate of a little over five percent. Despite this low unemployment rate there is a high proportion of low wage earners (22.5%), which is well above the European average (17.2%) (European Commission 2020), making the issue of fair and decent work a prominent issue in the German economy.

## Fairwork’s methodology

The Fairwork project evaluated the working conditions offered by 10 location-based^1^ digital labour platforms in Germany, by scoring them against the Fairwork principles and producing a national league table.

Our Fairwork principles (Fairwork 2020b) were co-developed in collaboration with the International Labour Organisation (ILO) and The United Nations Conference on Trade and Development (UNCTAD). We then held a number of workshops for local stakeholders in different countries. In Berlin, attendees included Berlin’s Senate Department for Labour and Social Affairs, the Federal Ministry of Labour and Social Affairs, and the German Trade Union Confederation (DGB). These workshops and related follow-up conversations with platform workers, policymakers, trade unions, platforms and labour lawyers allowed us to fine-tune the principles to the local German context.

The five Fairwork principles are:

- Fair Pay: workers should earn a decent income, after taking into account of work-related costs.
- Fair Conditions: platforms should protect workers from risks arising in the processes of work.
- Fair Contracts: contracts should be accessible, clear and transparent and should not put an undue liability on the worker.
- Fair Management: workers should be able to communicate effectively with a human representative of the platform and there should be due process for any decision affecting the worker. There should also be equity in the management process.
- Fair Representation: platforms should provide a documented process through which workers’ collective voice can be heard. Where unions are present, platforms should be willing to cooperate and negotiate with them.

For each of the five principles, a basic threshold and a more advanced threshold are established, so that each platform is scored out of 10. We assigned a score of 1 for each threshold only if we have enough evidence that the principle is satisfied. Therefore, a score of 0 may mean either that the threshold is not met, or that we did not have enough evidence to prove the platform is satisfying the principle. This allows us to score platforms that do not want to engage and share data with us.

In order to assign a score, Fairwork uses a qualitative threefold methodology, triangulating between three different sources of information (for a detailed discussion on the Fairwork methodology, see Fairwork 2021). The process starts with desk research in order to ascertain which platforms are operating in each city and to assess which are the largest and most influential ones. For the initial pilot year, only platforms operating in Berlin were selected to enter the league table. Desk research also gathers any publicly available information which could be used to score a platform.

Second, we interviewed platform managers to request evidence for each of the Fairwork principles. This allowed a more detailed understanding of the platform’s operations and working model. In case platform managers do not agree to engage with Fairwork researchers, the score only relies on the other two sources of information.

Third, for each platform we interviewed 6–10 workers directly. These interviews did not aim to create a representative sample but rather to understand the processes of work and the working conditions as experienced by the workers themselves. The interviews also allowed us to confirm or refute that policies and practices evidenced through the two other data collection methods are in place.

Final scores are then collectively decided by the researchers and are peer-reviewed by other members of the Fairwork team to ensure consistency across country scoring.

## Results: fairwork in Germany

Fairwork has so far released annual ratings for Germany, Ecuador, South Africa and India, with the findings reported below being based on the Fairwork annual report for Germany (Fairwork 2020a).

- **Fair Pay—**Contrary to many other countries of investigation, many platform workers in Germany in our sample are classified as employees. By being hired through an employment contract (rather than on a self-employment basis) workers are paid an hourly or monthly wage rather than on a piece-rate. Thus, unsurprisingly, of the 10 platforms investigated, all but one—Uber which relies on sub-contractors for its service—were able to evidence that workers receive at least €9.35 per hour which was the minimum wage in Germany in 2020. By having employment contracts, the majority of the digital platforms in our study were also able to evidence that workers earn the minimum wage even after work-related costs are taken into account.
- **Fair Conditions—**If classified as an employee, a platform worker in Germany is by law and national collective agreements entitled to a broad variety of working rights and protections, including full health and safety protection. Yet not all platforms in our sample could convincingly show to fulfill those standards—three failed to evidence that they protect their workers against occupational hazards. Amongst them is the ride-hailing platform Uber which operates its services through one carrier company which for its part engages other sub-contracting partners to perform the service. In consequence, most of the drivers are not directly engaged with Uber but rather by a sub-contracting company whose working conditions Uber does not consider or track. As a result of the articulated network of sub-contracting arrangements Uber could not show that the health and safety standards required by German law are unconditionally respected and fulfilled for its drivers.
- **Fair Contracts—**Rather than being subject to general terms and conditions, a large number of workers of digital platforms could rely on a formal employment contract. Employment contracts are strictly regulated by German labour law which sets distinct employment obligations and rights for both employees and employers. All employees in our sample as well as two self-employed workers stated that their employment contracts or terms and conditions respectively were easily accessible and written in plain language. This does not necessarily mean, though, that the existence of an employment contract or easy-to-access terms and conditions are well-known and understood amongst the platform workers. As a large part of platforms workers in Germany are migrants and potentially not proficient in German, as our study reveals, even clearly written contracts might not be understood by workers, who might therefore find it difficult to exercise their rights. Yet, in order to spread the knowledge of and bring to the attention the (sophisticated) legal formulations of German labour law both contracts and terms and conditions would need to be provided in the major migrant languages (e.g. Turkish, Spanish and Arabic) besides English—which is far from being the norm in Germany.
- **Fair Management—**Only five out of ten platforms could evidence to have a documented due process for decisions affecting workers such as disciplinary actions (e.g. suspensions) and deactivations. When it comes to preventing discrimination and promoting equity in the way workers are managed on a platform (in e.g. hiring or firing processes) and treated by customers and clients only one of the ten investigated platforms in Germany was able to evidence to have an appropriate policy in place—InStaff, a platform for the temporary recruitment of workers.
- **Fair Representation—**Freedom of association and worker voice mechanisms could be evidenced only by two out of ten of the digital platforms under investigation. ZenJob and Clevershuttle are the only ones in the sample recognizing a collective body that can undertake collective representation and bargaining. The green ride-pooling platform Clevershuttle, which was still operating in Berlin at the time of the investigation, was also in the process of establishing a work council in Berlin providing drivers with the possibility to exert their right of codetermination.

## Conclusion

Despite their recent arrival in Germany, platforms have already become a feature of the German economy, employing a rapidly increasing number of workers in many different sectors, including household services, logistics and transport. Both international and home-grown platforms have found in Germany a fertile ground for growth, but the spread of the platform economy has also been seen as contributing to undermining labour standards. Low and piece-rate pay, lack of health and safety protections, limited access to social protection, unclear and unintelligible contracts, lack of transparency in decision making, limited ability to be collectively represented are just some of the main issues platform workers face.

Although several policy initiatives have approached the issues associated with platform work, hitherto no major regulatory has been implemented, whilst social partners have had so far limited chance to intervene through collective bargaining. In this context, researchers at Fairwork have piloted for the first time the Fairwork project in Germany, in order to evaluate the labour standards offered by different digital platforms in the German platform economy.

10 platforms have been evaluated based on the five Fairwork principles: Fair Pay, Fair Conditions, Fair Contracts, Fair Management and Fair Representation. What emerged from the study is the fact that a number of platform workers are classified as employees rather than self-employed, and this guarantees a number of employment rights, including entitlement to minimum wage, health and safety protection and social protection. However, the existence of an employment relationship does not necessarily ensure platform work to be fair. For instance, contracts might not be transparent and clear to the workers, whilst collective representation among platform workers is still limited, buttressing an imbalance of power in the working relationship. Further, our study has revealed how the existence of complex network of sub-contracting can also contribute in diluting employment rights and in reducing the platforms’ accountability.

Policymakers are hence asked to mitigate between the fierce international competitive pressure in rapidly aging Germany which inevitably faces an increased shortage of skilled workers due to demographic reasons and thereby a net loss in its innovation ability and its historically rooted labour law and Tripartite Social Partnership established to ensure decent work and equal chances of social participation.

## Footnotes

1 Crowdwork platforms were not considered in this study.
